# Wavelet Analysis of Overnight Airflow to Detect Obstructive Sleep Apnea in Children

**DOI:** 10.3390/s21041491

**Published:** 2021-02-21

**Authors:** Verónica Barroso-García, Gonzalo C. Gutiérrez-Tobal, David Gozal, Fernando Vaquerizo-Villar, Daniel Álvarez, Félix del Campo, Leila Kheirandish-Gozal, Roberto Hornero

**Affiliations:** 1 Biomedical Engineering Group, University of Valladolid, 47011 Valladolid, Spain; veronica.barroso@gib.tel.uva.es (V.B.-G.); fernando.vaquerizo@gib.tel.uva.es (F.V.-V.); dalvarezgo@saludcastillayleon.es (D.Á.); fsas@telefonica.net (F.d.C.); robhor@tel.uva.es (R.H.); 2 Centro de Investigación Biomédica en Red en Bioingeniería, Biomateriales y Nanomedicina (CIBER-BBN), 47011 Valladolid, Spain; 3 Department of Child Health, The University of Missouri School of Medicine, Columbia, MO 65212, USA; gozald@health.missouri.edu (D.G.); gozall@health.missouri.edu (L.K.-G.); 4 Sleep-Ventilation Unit, Pneumology Department, Río Hortega University Hospital, 47012 Valladolid, Spain

**Keywords:** AdaBoost.M2, Bayesian multi-layer perceptron, airflow, children, obstructive sleep apnea, wavelet analysis

## Abstract

This study focused on the automatic analysis of the airflow signal (AF) to aid in the diagnosis of pediatric obstructive sleep apnea (OSA). Thus, our aims were: (*i*) to characterize the overnight AF characteristics using discrete wavelet transform (DWT) approach, (*ii*) to evaluate its diagnostic utility, and (*iii*) to assess its complementarity with the 3% oxygen desaturation index (*ODI*3). In order to reach these goals, we analyzed 946 overnight pediatric AF recordings in three stages: (*i*) DWT-derived feature extraction, (*ii*) feature selection, and (*iii*) pattern recognition. AF recordings from OSA patients showed both lower detail coefficients and decreased activity associated with the normal breathing band. Wavelet analysis also revealed that OSA disturbed the frequency and energy distribution of the AF signal, increasing its irregularity. Moreover, the information obtained from the wavelet analysis was complementary to *ODI*3. In this regard, the combination of both wavelet information and *ODI*3 achieved high diagnostic accuracy using the common OSA-positive cutoffs: 77.97%, 81.91%, and 90.99% (AdaBoost.M2), and 81.96%, 82.14%, and 90.69% (Bayesian multi-layer perceptron) for 1, 5, and 10 apneic events/hour, respectively. Hence, these findings suggest that DWT properly characterizes OSA-related severity as embedded in nocturnal AF, and could simplify the diagnosis of pediatric OSA.

## 1. Introduction

Pediatric obstructive sleep apnea (OSA) is a major sleep-related breathing disorder, affecting a large number of children (5%) and increasing the risk of negative health consequences among those affected [1,2]. Several studies have reported that the characteristic nocturnal respiratory disruptions manifesting as either cessation or reductions in airflow lead to inadequate gas exchange and fragmented sleep that affects not only other physiological processes, but also cognitive development [2,3,4]. Consequently, the morbidities can worsen and become irreversible if OSA is not timely treated [5].

Despite the potential seriousness of OSA-related complications, it remains an underdiagnosed disorder due to relative unawareness of both parents and primary care physicians and the inherent difficulties in accessing the diagnostic test [6,7]. In this regard, the gold standard approach for diagnosing OSA is overnight polysomnography (PSG), which is technically complex, labor intensive, expensive, potentially distressing to the child and uncomfortable to the parent, and relatively unavailable [6,7]. These drawbacks have led to multiple efforts focused around development of alternative methods that simplify OSA diagnosis in children. Typically, these studies centered around the analysis of a reduced number of physiological signals, such as photoplethysmography (PPG), electrocardiogram (ECG), airflow (AF), or blood oxygen saturation (SpO_2_), using statistical, nonlinear and/or spectral techniques [8,9,10,11,12,13,14]. However, most of these analyses are a direct extension of techniques previously used in studies of OSA in adults, and have not achieved the same efficacy metrics so far [8,9,10,11,12,13,14]. Major reasons for such differences reside in the fact that the density of respiratory events is remarkably lower in children, and the criteria to determine the severity categories are more conservative in children [1,5,15], thereby presenting greater difficulty for automatic analyses. Consequently, different approaches are required in order to detect and characterize the disturbances that the presence of OSA entails during physiological recordings in children.

In this study, we propose the analysis of oronasal airflow to enable the diagnosis of pediatric OSA, in light of the fact that respiratory events are defined based on total/partial reduction (apneas/hypopneas) of airflow [15]. Overnight AF tracings reflect the respiratory activity of children while asleep, including the cessations/reductions of AF in the context of OSA [15]. Unsurprisingly, AF analytics have already shown their utility in simplifying careening and potentially the diagnosis of pediatric OSA [11,12,16,17]. In addition, apneic events are usually, albeit not always accompanied by blood oxygen desaturations [15]. Consequently, conventional oximetry indices, such as the oxygen desaturation index ≥3% (*ODI*3), are typically used as a suboptimal surrogate of PSG when the latter is unavailable [18,19]. However, these indices routinely underestimate the severity of the disease [20,21], thus requiring complementary approaches.

AF is a non-stationary biomedical signal, that is, its properties change and evolve over time [17]. Thus, we propose implementation of wavelet analysis of AF. Unlike the methods based on Fourier transform, the wavelet transform (WT) does not make assumptions about the stationarity of the time series [22,23]. Hence, the WT decomposition is a promising method for the analysis and characterization of AF signal, which overcomes the limitations of conventional spectral analyses. In addition, WT analysis is capable of providing optimal time-frequency resolution (high time and frequency resolution at high and low frequencies, respectively), as well as high frequency resolution in long-lasting recordings [22,24]. It should be noted that a high frequency resolution at low frequencies is of utmost importance for the current study, since previous research has reported the existence of relevant OSA-related information at low frequencies of the AF signal [12,16]. Moreover, this property would allow us to obtain detailed information about a certain frequency range, while preserving the temporal information related to apneic events [25]. This methodology has been used successfully to characterize other signals involved in OSA diagnosis in adults and children, such as the electroencephalogram [26], ECG [27], SpO_2_ [25], and thoracic or abdominal effort [28,29]. However, WT analysis will be to characterize nocturnal AF in the context of pediatric OSA for the first time. Therefore, our starting hypothesis is that WT can provide useful information about the AF behavior in the presence of apneic or hypopneic events. Accordingly, the aims of this study were: (*i*) to characterize nocturnal AF by means of WT, (*ii*) to assess its utility to diagnose OSA in children, and (*iii*) to assess its complementarity with *ODI*3.

## 2. Database

Children (946; 584 males and 362 females), clinically suspected of suffering from pediatric OSA, spent one night in the specialized sleep laboratory of the Comer Children’s Hospital of the University of Chicago (Chicago, IL, USA) to undergo nocturnal polysomnography. The children’s caretakers were informed and agreed to participate in the study. The protocol was approved by the Ethics Committee of the University of Chicago (approval numbers: 11-0268-AM017, 09-115-B-AM031, and IRB14-1241). 

During the PSG, up to 32 physiological signals were recorded, including AF. Following the guidelines of the American Academy of Sleep Medicine (AASM) [15], sleep medicine specialists scored the apnea and hypopnea events from these recordings to calculate the apnea-hypopnea index (AHI: number of apneic and hypopneic events per hour of sleep). Each child was then classified into one of the following four severity categories: AHI ∈ [0,1) events/hour (e/h) as no-OSA, AHI ∈ [1,5) e/h as mild OSA, AHI ∈ [5,10) e/h as moderate OSA, and AHI ∈ [10,∞) e/h as severe OSA [1,5].

The main characteristics of the children involved in the study are presented in Table 1. Out of the 946 children, 570 (60%) were randomly selected into the training group and 376 (40%) into the test group. Statistically significant differences (*p*-value < 0.01) between the training and test groups were evaluated using Fisher’s exact test for categorical variables and the non-parametric Mann-Whitney *U* test for continuous variables. No significant differences were found in any of the evaluated clinical or demographic variables.

AF and SpO_2_ recordings were obtained from the PSG recordings by means of a thermistor and a pulse oximeter, respectively. According to AASM, the AF and SpO_2_ recordings used in our study were resampled at the recommended frequency: 100 Hz for AF and 25 Hz for SpO_2_ [15]. Both signals were subjected to a preprocessing stage in order to automatically remove possible artifacts. This stage was conducted following the artifact removal methods proposed in previous studies [12,17]. Signals whose duration was less than 3 h after artifact removal were excluded from our study [17,30]. Moreover, AF signals were normalized to minimize the inter-individual differences related to particular physiological characteristics other than OSA [31].

## 3. Methods

**Figure 1:**
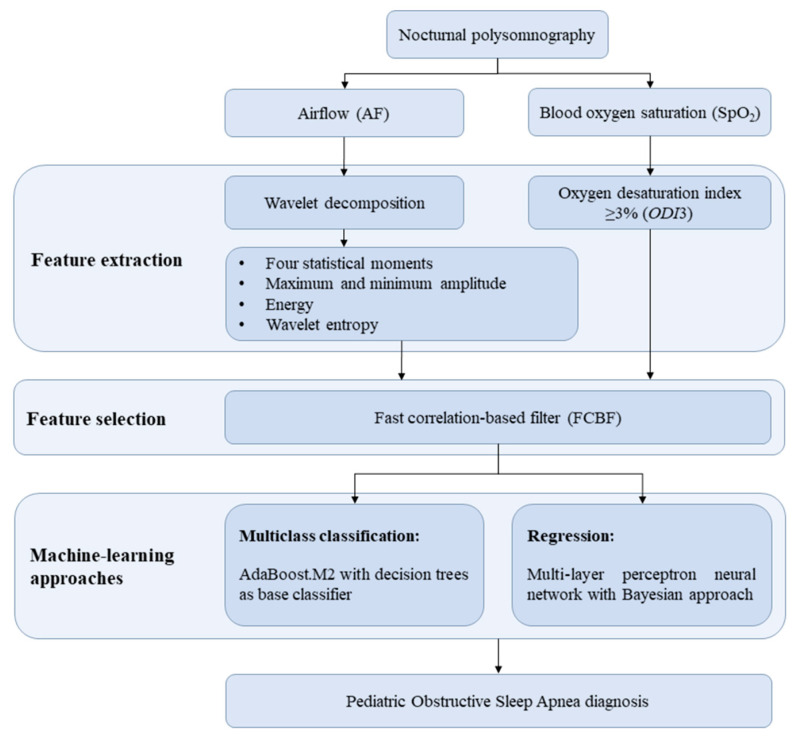
Scheme of the proposed methodology.

Figure 1 shows the scheme of the stages followed in this study: (*i*) feature extraction to characterize AF by means of WT, as well as to compute *ODI*3, (*ii*) feature selection using the fast correlation-based filter (FCBF) to obtain an optimal feature subset, and (*iii*) application of machine-learning approaches to carry out multiclass classification and regression. The multiclass classification was performed to determine the OSA severity degree through AdaBoost.M2 with decision trees as base classifier. Regarding to the regression process, it was carried out to estimate the AHI of each child by means of multi-layer perceptron neural network with a Bayesian approach (BY-MLP). In this sense, recent studies have shown the usefulness of these selection and machine-learning methods in the context of pediatric OSA diagnosis [12,17].

### 3.1. Feature Extraction

#### 3.1.1. Wavelet Features

**Figure 2:**
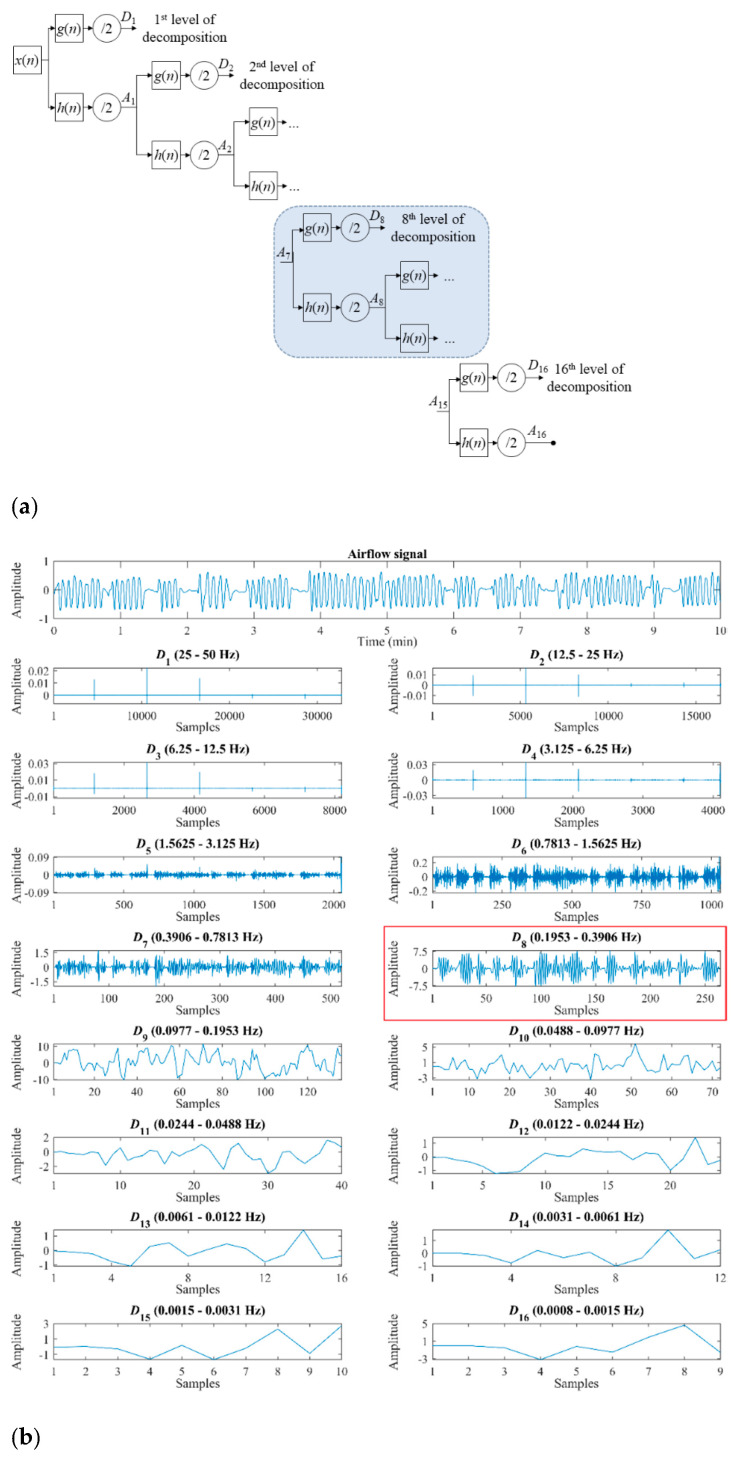
(**a**) Wavelet decomposition where *x*(*n*) is the time series, *g*(*n*) are half-band high-pass filters, *h*(*n*) are half-band low-pass filters, /2 are subsampling processes, and *D_j_* and *A_j_* are detail and approximation signals of the decomposition level *j*, respectively. (**b**) Detailed signals at each decomposition level of airflow signal.

WT allows to conduct a multiresolution analysis of non-stationary signals, i.e., it allows to analyze a time series in different frequency ranges with variable resolution [24]. In our study, the analysis is performed using the discrete wavelet transform (DWT) to deal with the redundancy and computational complexity issues of the continuous approach [23,24]. Moreover, DWT has been successfully used in previous studies aimed at OSA detection [25,26,28,29]. As can be seen in Figure 2, multiresolution analysis with DWT consists of performing *N* = log_2_(*M*) decomposition steps, where *M* is the size of the time series *x*(*n*) [23,25]. At each step, the decomposition is conducted by using a scaling *ϕ_j,n_* and a wavelet function *ψ_j,n_*, which are generated by scaling and translation of basis functions and where *j* denotes the decomposition level [32]:

(1) $$\phi_{j,k}\left(n\right)=s^{-\frac{j}{2}}\cdot \phi \left(\frac{n-k\cdot \tau \cdot s^{j}}{s^{j}}\right)=2^{-\frac{j}{2}}\cdot \phi \left(2^{-j}\cdot n-k\right),$$

(2) $$\psi_{j,k}\left(n\right)=s^{-\frac{j}{2}}\cdot \psi \left(\frac{n-k\cdot \tau \cdot s^{j}}{s^{j}}\right)=2^{-\frac{j}{2}}\cdot \psi \left(2^{-j}\cdot n-k\right),$$

where *s* = 2 and *τ* = 1 (dyadic sampling) are the scale and translation parameters, respectively, *j* ∈ Z is the decomposition level, *k* ∈ Z is the coefficient position within each sub-band of the decomposition, *ϕ* is the basis scaling function, and *ψ* is the basis wavelet function or mother wavelet. The scaling and wavelet functions allow to characterize the approximation and detail spaces at different resolutions, respectively. It cannot be affirmed that a generic optimal wavelet function exists, since in each particular case there will be a mother wavelet that better adapts to the signal under study. 

**Figure 3:**
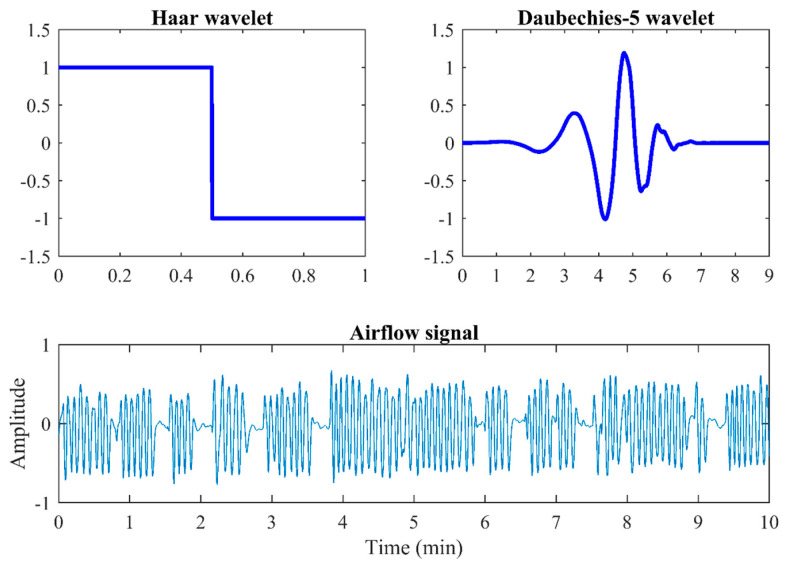
Mother wavelets Haar and Daubechies-5, and a 10-min segment of AF signal.

In our work, Haar and Daubechies-5 wavelets have been evaluated due to their previous suitability to the AF signal [33,34]. Figure 3 shows the mother wavelets Haar and Daubechies-5, as well as a 10-min segment of AF signal. Haar is the simpler orthonormal wavelet, which does not cause edge effect due to it uses a single vanishing moment and a support width = 1 (Figure 3) [35,36]. It would avoid ignoring the relevant information contained in these regions. Moreover, the stepped shape of the Haar wavelet would allow to detect abrupt reductions of AF caused by apneic events. Regarding Daubechies-5, it uses 5 vanishing moments and a support width = 9 (Figure 3). When increasing the vanishing moment and the support width, the time-frequency localization improves [37,38], but the edge effect also increases [36]. Hence, the Daubechies-5 wavelet would provide a better localization while causing less edge effect than other higher order variants, such as Daubechies-10 or Daubechies-20 [36]. In addition, its shape resembles an AF signal, which would allow a better setting.

In practice, the scaling (*ϕ*) and wavelet (*ψ*) functions are considered as half-band low-pass *h*(*n*) and half-band high-pass *g*(*n*) filters, respectively, such as [24,32]:

(3) $$g\left(n\right)={\left(-1\right)}^{1-n}\cdot h\left(1-n\right).$$

Hence, as shown in Figure 2a, DWT can be implemented as a cascade of recursive filters, each of them followed by a subsampling process by 2 (dyadic sampling) to reduce the sampling frequency and increase the spectral resolution [24,29,32]. Thereby, the low frequency band can be split again, thus generating new levels of decomposition. At each decomposition level *j*, after the corresponding filter and subsampling, the approximation *A_j_* (low frequency) and the detail *D_j_* (high frequency) signals are obtained [24,25,29]:

(4) $$A_{j}\left[k\right]=\sum_{n}A_{j-1}\cdot h\left(2k-n\right),$$

(5) $$D_{j}\left[k\right]=\sum_{n}A_{j-1}\cdot g\left(2k-n\right),$$

where *A*_0_ is the time series *x*(*n*). This decomposition process finishes when level *j* = *N* is reached.

The AF signal was segmented into epochs of length 2^16^ samples (≈10-min), as conducted in previous studies [12,39]. Thus, DWT decomposition for each segment was carried out at 16 levels (*N* = log_2_ (2^16^)), using Haar and Daubechies-5 as mother wavelets [33,34]. An example of decomposition of AF segment by means of Daubechies-5 can be observed in Figure 2b.

Our DWT analysis mainly focused on *D*_8_ (0.1953–0.3906 Hz), which corresponds to the normal breathing frequency band in sleeping children [16,40]. This choice let us characterize the alterations caused by OSA in normal nocturnal respiration. Thereby, after obtaining the detail coefficients of *D*_8_ from each of the 946 AF signals, the following features were extracted to quantify the information contained in them [25]:

- Four statistical moments (*M*_1*D*8_–*M*_4*D*8_). Mean (*M*_1*D*8_), standard deviation (*M*_2*D*8_), skewness (*M*_3*D*8_), and kurtosis (*M*_4*D*8_) are computed to measure central tendency, dispersion, asymmetry, and peakedness of the distribution of the coefficients of *D*_8_ [25].
- Maximum and minimum (*Max_D_*_8_ and *Min_D_*_8_). They are the highest (*Max_D_*_8_) and the lowest (*Min_D_*_8_) value of the coefficients of the detail signal *D*_8_. These features allow to quantify the maximum and minimum amplitude reached in this decomposition level [25].
- Energy (*E_D_*_8_). This feature measures the quadratic amplitude of the detail signal *D*_8_, providing information about the activity produced in the resolution level associated to the representative frequency band of the normal breathing [25,41]. It is computed as the sum of the modulus of the detail coefficients squared [22,23]:
  (6) $$E_{D8}=\sum_{k}{\left|D_{8}\left[k\right]\right|}^{2},$$

In addition to the *D*_8_-derived features, the wavelet entropy from all detail levels has been obtained to also characterize the OSA global effects on the complete AF signal:

- Wavelet entropy (*WE*). It is an extension of the well-known Shannon’s entropy. Therefore, this feature allows quantifying the energy distribution changes generated in the decomposition process, offering information about the underlying dynamical behavior and the irregularity of the signal [22,25,41]:
  (7) $$WE=-\sum_{j=1}^{N}p_{j}\cdot \log\left(p_{j}\right),$$
  where *p_j_* is the normalized energy distribution at the decomposition level *j*:
  (8) $$p_{j}=\frac{E_{Dj}}{\sum_{j=1}^{N}E_{Dj}}\;.$$

Due to the amplitude reductions of the AF signal produced by apneic events [15], as well as the ability of the DWT to assign low coefficients to the flatter signal parts and high coefficients to the steeper [42], it is expected to find higher coefficients in the signal *D*_8_ of the subjects without OSA. Consequently, and according to previous spectral analyses of AF [16,43], higher values of *M*_1*D*8_, *M*_2*D*8_, *Max_D_*_8_, and *Min_D_*_8_, as well as lower values of *M*_3*D*8_ and *M*_4*D*8_, are expected in these subjects. In addition, it is also expected that there will be greater activity in the normal breathing band in the absence of apneas and hypopneas, i.e., that subjects without OSA have a higher *E_D_*_8_. Regarding *WE*, it has been observed that apneic events introduce changes in time and frequency domains of AF signal of children [11,12,16]. Thus, AF signals are expected to be more irregular (higher values of *WE*) as OSA severity increases. Thereby, since DWT allows to obtain higher frequency resolution at low frequencies, it is expected to provide a more detailed analysis than classical spectral analysis.

#### 3.1.2. Oximetry Index

*ODI*3 was obtained from the SpO_2_ signal in order to compare the AF information with a common clinical index that usually acts as a surrogate of the full PSG, when the latter is not available. The guideline followed to calculate *ODI*3 was established according to the definition provided by Taha et al. [44]. Thus, the SpO_2_ reductions greater than or equal to 3% and lasting at least 10 s were considered desaturation events. Finally, *ODI*3 was computed dividing the total number of desaturations presented in the SpO_2_ signal by the recording time expressed in hours. Due to the blood oxygen desaturations are closely related to the occurrence of apneic events [15], *ODI*3 is expected to be higher as OSA severity increases.

### 3.2. Feature Selection

The fast correlation-based filter (FCBF) was used to obtain an optimal subset of relevant and non-redundant features [45]. Some motivations for using this algorithm are that it does not depend on subsequent analyzes and it reduces the complexity and dimensionality of the predictive models [45,46]. Moreover, this method has been successfully applied in the pediatric OSA context [12,13,17,25,47].

A bootstrapping procedure was conducted (1000 replicates) to obtain a stable and optimal subset [46]. The average significance was used as selection threshold *T_s_* [30]. It was computed as the average number of times that all features were selected [30]. Hence, the features selected a number of times ≥*T_s_* constituted the optimal set of features that fed the predictive models proposed in our study.

### 3.3. Machine-Learning Approaches

#### 3.3.1. Multiclass Classification

In order to carry out a classification into 4-classes (no-OSA, mild, moderate, and severe OSA), we used the adaptive boosting (AdaBoost.M2) ensemble-learning algorithm due to previous success in classifying AF signals in the OSA context [12,48]. AdaBoost.M2 is based on iterative training of multiple ‘weak classifiers’ (also, base classifiers) of the same type, so that each new one focuses on the data misclassified by its predecessors [49,50]. Thereby, this method calls the base classification algorithm *L* times, giving it each time a different weight distribution for the training. The same weight is initially assigned to all instances and then it is updated in each iteration: more weight to wrongly classified instances and less weight to those correctly classified [49,50]. This allows the algorithm to adapt to the data, minimizing the expected error and focusing on correctly classifying the instances with more weight [50]. The weight update also involves the use of the learning rate *α*, a regularization parameter to deal with overfitting [48,50]. Finally, the weighted vote of all the previously trained classifiers is computed, obtaining a more robust prediction of each class [49,50].

In our study, decision trees were used as base classifiers, which is a common choice when using ensemble-learning methods [49]. The optimal number of weak classifiers *L*, as well as the learning rate *α*, require to be tuned. In this regard, both hyperparameters were optimized by applying bootstrapping (1000 replicates) in the training group and using 0.632 bootstrap to estimate the Cohen’s kappa (kappa) for each *L*/*α* pair [12,48].

#### 3.3.2. Regression

A regression process by means of multi-layer perceptron neural network with a Bayesian approach (BY-MLP) was conducted to estimate AHI. BY-MLP has already shown its utility in OSA diagnosis [17,51,52]. This method is based on a set of perceptrons organized in layers, so that each perceptron is connected to all those of the next layer with a certain weight [53]. In order to obtain a universal approximation, BY-MLP is typically formed by 3 layers: (*i*) an input layer with *N_I_* perceptrons that receive the input patterns and propagate them to all the perceptrons of the next layer, (*ii*) a hidden layer with *N_H_* perceptrons that perform a non-linear processing of the received patterns and propagate it to the next layer, and (*iii*) an output layer with *N_O_* perceptrons that process the information received from the hidden layer and provide the response of the neural network [53]. The learning process of the network involves adjusting the weights associated to the connections between perceptrons. The technique applied to carry out this adjustment is the Bayesian inference, which allows finding the optimal weights that minimize the error function [52].

In our study, *N_I_* is equal to the number of features selected in the previous stage, *N_H_* is a parameter to be tuned, and *N_O_* is equal to one perceptron since the network purpose is to estimate the AHI. As in Adaboost.M2, the value of *N_H_* was optimized by applying bootstrapping (1000 replicates) in the training group and using 0.632 bootstrap to estimate the kappa [51].

### 3.4. Statistical Analysis

The data distribution of each biomedical index was evaluated by means of the Lilliefors test. The results showed that wavelet features did not follow a normal distribution. Consequently, the existence of statistically significant differences (*p*-value < 0.01) among OSA severity groups was assessed by means of the non-parametric Kruskal-Wallis test. Moreover, Spearman’s correlation was used to evaluate the relationship between AHI and the features under study. Cohen’s kappa of two-class (kappa_2_) and four-class (kappa_4_), as well as the four-class accuracy (Acc_4_), assessed the agreement between predicted and actual diagnosis [54]. In addition, the metrics used to evaluate the diagnostic performance of the machine-learning approaches for the common AHI thresholds 1 e/h, 5 e/h, and 10 e/h were sensitivity (Se), specificity (Sp), accuracy (Acc), positive and negative predictive values (PPV and NPV, respectively), and positive and negative likelihood ratios (LR+ and LR-, respectively). The statistically significant differences (*p*-value < 0.001) between diagnostic metrics of the models were evaluated using the Mann-Whitney *U* test for pairwise comparison with Bonferroni correction.

## 4. Results

### 4.1. Training Group

#### 4.1.1. Extracted Features

The coefficients of *D*_8_ with and without sign computed by means of Haar obtained features with an average Spearman’s correlation of 0.2075 and 0.3190, respectively. Regarding Daubechies-5, the features achieved an average correlation of 0.2379 and 0.3447 for coefficients with and without sign, respectively. Consequently, the features extracted from *D*_8_ in absolute value and with Daubechies-5 were used in the following stages of our study.

**Figure 4:**
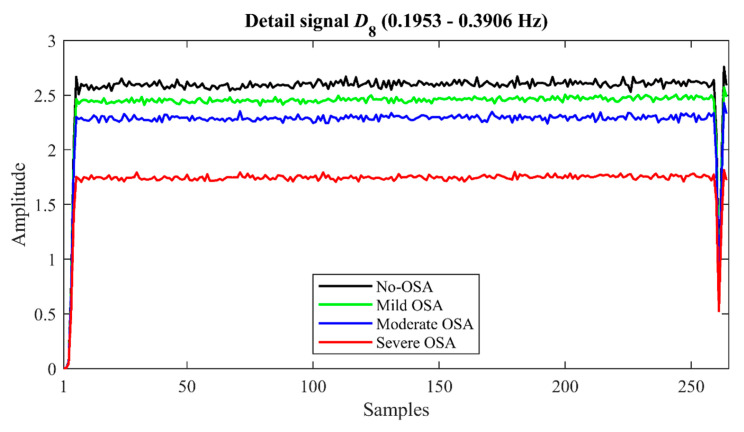
Averaged *D*_8_ signal by OSA severity groups in the training group.

**Figure 5:**
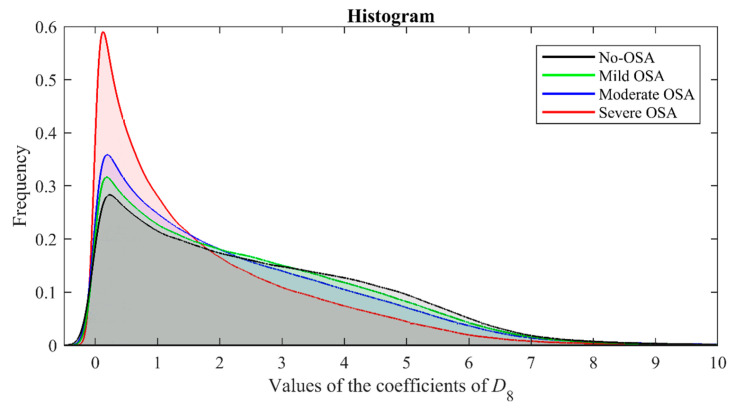
Distribution of the coefficients of *D*_8_ by OSA severity in the training group.

Figure 4 displays the averaged detail signal *D*_8_ for each severity group in the training dataset. In this figure, higher amplitude values of *D*_8_ can be observed in subjects without OSA. In addition, the frequency distribution of the values of the *D*_8_ coefficients as OSA severity increases can be visualized in Figure 5. According to this figure, coefficients close to 0 are more frequent in children with OSA, increasing the asymmetry and the sharpness of the peak of the distribution as the AHI increases.

Table 2 shows the median and interquartile range values by OSA severity group of each feature, as well as its Spearman’s correlation coefficient with the AHI and the *p*-value obtained by means of the Kruskal-Wallis test in the training group. *M*_1*D*8_, *M*_2*D*8_, *Max_D_*_8_, *Min_D_*_8_, and *E_D_*_8_ showed a decreasing trend as OSA severity increases. In contrast, the tendency of *M*_3*D*8_, *M*_4*D*8_, and *ODI*3 was towards higher values. Regarding *WE*, it showed less separability between groups, although with a notable increase in the most severe subjects. In addition, all the extracted features showed significant differences among OSA severity groups (*p*-value < 0.01 after Bonferroni correction).

#### 4.1.2. Feature Selection

**Figure 6:**
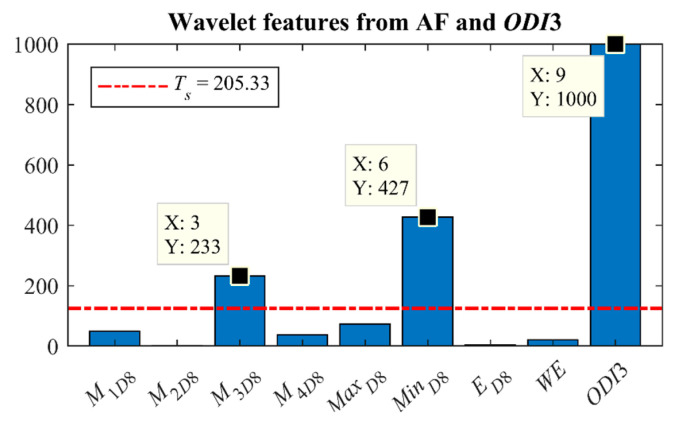
Results of feature selection using FCBF in 1000 bootstrap from the training group with wavelet features from AF and *ODI*3.

We carried out 2 selection trials, one only with wavelet features from AF and another that also included the *ODI*3. FCBF was applied to 1000 bootstrap replicates obtained from the training group. In the trial with wavelet features from AF, only *M*_3*D*8_ was selected more than *T_s_* times (*T_s_* = 125.25). As can be seen in Figure 6, *M*_3*D*8_, *Min_D_*_8_, and *ODI*3 exceeded this threshold (*T_s_* = 205.33) when *ODI*3 was included in the selection process.

#### 4.1.3. Optimization of Adaboost.M2 and BY-MLP

Two Adaboost.M2 models (AB^AF^ and AB^AF,*ODI*3^) and two BY-MLP models (BY-MLP^AF^ and BY-MLP^AF,*ODI*3^) were designed and trained after the feature selection stage. AB^AF^ and BY-MLP^AF^ models were fed only with the selected wavelet feature from AF (*M*_3*D*8_), while AB^AF,*ODI*3^ and BY-MLP^AF,*ODI*3^ models incorporated wavelet features from AF (*M*_3*D*8_ and *Min_D_*_8_) and *ODI*3. Regarding the Adaboost.M2 models, we conducted trials with values of *L* = [1:9 10:10:90 100:100:900 1000:1000:10000] and of *α* = [0.1:0.1:1]. The optimization of these parameters was based on the maximum kappa obtained through 0.632 bootstrap for the *L*/*α* pair: *L* = 8000 and *α* = 1 for AB^AF^ and *L* = 3000 and *α* = 1 for AB^AF,*ODI*3^. Regarding the BY-MLP models, values of *N_H_* = [1:1:40] were used. In this case, the maximum kappa was reached with *N_H_* = 1 for BY-MLP^AF^ and *N_H_* = 36 for BY-MLP^AF,*ODI*3^.

### 4.2. Test Group

In order to improve the generalization of our results, the trained models were assessed in 1000 bootstrap replicates from the test group. The diagnostic performance metrics were obtained by means of the bootstrap 0.632 procedure and the statistically significant differences (*p*-value < 0.001) between models were evaluated using the Mann-Whitney *U* test for pairwise comparison with the Bonferroni correction. Table 3 and Table 4 show the diagnostic performance (median [95% confidence interval]) achieved by each of the machine-learning models proposed in our study, as well as the *ODI*3. *ODI*3 showed a severity underestimation in 1 and 5 e/h. Regarding AB^AF^ and BY-MLP^AF^ models, these obtained an unbalanced Se-Sp pair, with a severity overestimation in 1 and 5 e/h and an underestimation in 10 e/h. When combining wavelet features from AF with *ODI*3 (AB^AF,*ODI*3^ and BY-MLP^AF,*ODI*3^) these negative effects of overestimation and underestimation were reduced. BY-MLP^AF,*ODI*3^ obtained highest Acc for 1 and 5 e/h and AB^AF,*ODI*3^ for 10 e/h, significantly outperforming (*p*-value < 0.001) the individual approaches. Moreover, these models also achieved higher performance than AB^AF^, BY-MLP^AF^, and *ODI*3 in terms of kappa_2_, kappa_4_, and Acc_4_.

## 5. Discussion

In the present work, we characterized overnight pediatric AF by means of wavelet features obtained from the normal respiratory band. In addition, we showed the complementarity between the wavelet analysis conducted on AF and *ODI*3, obtaining two machine-learning models with high performance to diagnose pediatric OSA. The interpretation of our findings is detailed below.

### 5.1. Training Group

Our results revealed that the mother wavelet Daubechies-5 is better suited to the nocturnal AF behavior of children than the Haar wavelet. This may be because Daubechies-5 uses more vanishing moments and a larger support width than Haar (Figure 3), which allowed to obtain a more precise phase space and time-frequency location, as well as to focus and detect the singularities of the AF signal [37,38].

According to Figure 4 and Figure 5, the subjects without OSA presented higher values of *D*_8_, as well as a less asymmetric and less peaked distribution than the subjects with OSA. These amplitude and distribution differences agree with the information provided by the statistical analysis carried out in the training group. Thereby, *M*_1*D*8_ and *M*_2*D*8_ showed a decreasing tendency as the OSA severity increased. This fact is consistent with a less activity in the normal breathing band as AHI increased, which causes a notable reduction of the coefficients of signal *D*_8_ from AF and a narrower dispersion range. Regarding *M*_3*D*8_ and *M*_4*D*8_, these experienced an increasing tendency, i.e., greater positive skewness and greater sharpness of the distribution peak in lower values of the coefficients of *D*_8_ as the AHI is higher. This indicates that apneas and hypopneas change the frequency distribution of AF signal and reduce its frequency components in the normal breathing band, which leads to fewer high coefficients and more coefficients close to zero in this band. According to this reduction of coefficients, the maximum and minimum values of *D*_8_ (*Max_D_*_8_ and *Min_D_*_8_), as well as the energy of this level (*E_D_*_8_), were lower as the severity of OSA increased. This decrease revealed that apneic events reduce the detail signal amplitude and the activity produced in the resolution level associated to the normal breathing band, which agrees with a lower occurrence of normal breathing patterns. Moreover, an increase of *WE* could also be observed in the severely affected children. This fact suggests that severe OSA disturbs the energy distribution of AF signal at the different levels of decomposition. In this way, the wavelet energy is redistributed in other frequency bands associated with apneic events instead of concentrating in the normal breathing band. Consequently, the AF signal becomes more irregular in these cases. Thus, we showed that DWT provided useful information to characterize the nocturnal AF of children, as well as its behavior in presence of apneic events.

### 5.2. Feature Selection and Diagnostic Performance

Despite the clear tendencies shown by the 8 wavelet features extracted from AF, only the asymmetry of the distribution of the coefficients of *D*_8_ (*M*_3*D*8_) was relevant and not redundant with respect to the rest. This is coherent with the statistical analysis carried out in the training group, where *M*_3*D*8_ not only showed significant differences among severity groups, but was also the wavelet feature that obtained highest Spearman’s correlation with the AHI. Thus, the selection of *M*_3*D*8_ suggests that its individual utility to characterize the pediatric OSA is greater than individual and joint usefulness of the other wavelet features.

However, FCBF revealed that there is complementarity between *ODI*3 and the wavelet information obtained from both *M*_3*D*8_ and *Min_D_*_8_. In this regard, *M*_3*D*8_, *Min_D_*_8_, and *ODI*3 showed a clear separability among OSA severity groups (lower *p*-value), as well as a higher Spearman’s correlation with the AHI. Notice that FCBF did not select *Min_D_*_8_ in the trial with wavelet features from AF, but it was selected when *ODI*3 was included in the selection process. Due to the final subset is obtained as the union of the feature subsets selected in ≥*T_s_* bootstrap replicates [46], this fact suggests that *Min_D_*_8_ contributes with additional information to *ODI*_3_ and different from that provided by *M*_3*D*8_, which highlights the joint usefulness of these features. Therefore, the information about the occurrence of apneic events provided by DWT through the distribution asymmetry (*M*_3*D*8_) and the minimum amplitude of *D*_8_ (*Min_D_*_8_) from AF is complementary to the information provided by *ODI*3 about the occurrence of desaturations.

This complementarity was also reflected in the machine-learning models used to classify children in 4-classes of OSA severity and estimate their AHI. AB^AF^, BY-MLP^AF^, and *ODI*3 achieved a moderate diagnostic performance in the testing group. These approaches were significantly outperformed (*p*-value < 0.001) by BY-MLP^AF,*ODI*3^ in kappa_2_ for the 3 AHI cut-offs, kappa_4_, and Acc_4_, as well as by AB^AF,*ODI*3^ in kappa_2_ for 1 and 10 e/h and kappa_4_. This fact revealed that the agreement between the predicted and actual OSA severity improves when wavelet information and *ODI*3 are combined, which confirmed the complementarity of both approaches. Moreover, the diagnostic accuracies reached by BY-MLP^AF,*ODI*3^ for 1, 5, and 10 e/h and AB^AF,*ODI*3^ for 1 and 10 e/h were also significantly higher (*p*-value < 0.001) than those obtained by the individual approaches. In this regard, BY-MLP^AF,*ODI*3^ achieved a more balanced Se-Sp pair in 5 e/h, as well as significantly higher Se and Acc (*p*-value < 0.001) than AB^AF,*ODI*3^ in 1 and 5 e/h. Hence, the AHI estimated by BY-MLP^AF,*ODI*3^ could be used by medical personnel to discriminate between mildly to moderately affected subjects. In addition, AB^AF,*ODI*3^ obtained a statistically significant higher Acc (*p*-value < 0.001) for 10 e/h. Furthermore, as LR+ > 10 is a robust indicator to determine the presence of a disease [55], the significantly higher LR+ (*p*-value < 0.001) obtained with AB^AF,*ODI*3^ for 10 e/h (LR+ = 18.99 [14.60, 51.76]) indicated that this model provides greater evidence than BY-MLP^AF,*ODI*3^ to detect severe OSA. Consequently, this multiclass classifier could be used as a potential automatic tool to this purpose. Therefore, the methodology proposed in the present study would be a useful alternative to nocturnal polysomnography, since it would help to simplify and streamline the pediatric OSA diagnosis in a timely fashion that potentially prevents the generation and worsening of deleterious consequences.

### 5.3. Comparison with Other Studies

As shown in Table 5, several studies focused on the simplification of pediatric OSA have applied automatic analysis techniques to polysomnographic signals such as ECG, PPG, SpO_2_, and AF [8,9,10,11,12,13,14,17,25,47]. In this regard, Shouldice et al. [10] applied temporal and spectral analysis methods to 50 ECG signals, obtaining 84.00% Acc for 1 e/h. Other studies such as those carried out by Gil et al. [8] and Dehkordi et al. [9] analyzed 21 and 146 PPG signals, respectively. They evaluated their proposal using 5 e/h as AHI cut-off for positive OSA and reached 80.00% Acc and 71.00% Acc, respectively. However, despite the high diagnostic performance achieved by these studies, the small sample size makes their results difficult to generalize.

In contrast, some studies involved a larger number of children (207–4191) [12,13,14,47]. Hornero et al. [13], Xu et al. [47], and Garde et al. [14] based their studies on the automatic analysis of SpO_2_ signals, while Jiménez-García et al. [12] focused on AF together with SpO_2_. They used the AHI cut-offs 1, 5, and 10 e/h, obtaining accuracies that ranged between 75.00–81.28%, 79.40–82.05%, and 88.19–90.26%, respectively. However, the diagnostic accuracies obtained by these studies were outperformed by our current proposal for all the 3 AHI cut-offs.

The work of Vaquerizo-Villar et al. [25], a previous study from our research group, was based on wavelet analysis combined with other features extracted from 981 SpO_2_ signals. This study used only the AHI cut-off 5 e/h to determine the presence of OSA, achieving a high Acc. However, our current proposal was evaluated according to the different severity categories, which allowed us to also identify children without OSA as well as the severely affected ones. In this regard, our methodology showed great usefulness for detecting severe cases (Acc = 90.99% [89.29,92.61] and LR+ = 18.99 [14.60,51.76] with AB^AF,*ODI*3^ for 10 e/h). As these pediatric subjects have an increased risk of developing comorbidities and neurocognitive deficits [1,5], our tool would allow to diagnose and treat these cases early on, before the consequences are irreversible. Moreover, automatic detection of no-OSA cases would reduce the waiting lists and streamline the diagnosis of children affected by this disease. In addition, it is worth noting the achieved improvement with respect to our previous study based on recurrence plots from AF [17]. Although the Acc in 1 e/h was lower, a more balanced Se-Sp pair should be noted, as well as the higher diagnostic performance obtained in 5 e/h. Moreover, we outperformed the Acc and LR+ obtained in 10 e/h, thus our current proposal is more robust to detect severe OSA. Regarding our latest work focused on the bispectral analysis of AF [56], it was improved in Sp, PPV, and LR+ for 1 and 5 e/h, as well as in all diagnostic metrics for 10 e/h by the current proposal. Hence, the DWT would be a more potentially useful tool than bispectrum to diagnose severe OSA cases in a timely fashion. Furthermore, DWT revealed changes of energy and frequency components of AF, that could not have been detected with bispectrum or recurrence plots.

### 5.4. Limitations and Future Work

One of the limitations of this study is the size of the population under study, since it would have been desirable to analyze a larger set of AF signals originating from multiple sources to ensure more generalizable results. Although we have shown that Daubechies-5 better reflects the behavior of AF than Haar, other mother wavelets that have not yet been applied in this context could be analyzed in future studies, and the results obtained with each of them could be compared. Moreover, it would also be interesting to search for mother wavelets that more closely resemble the shape of AF signal. The use of DWT and the analysis of the detail level *D*_8_ have also shown its usefulness in the diagnosis of the disease. However, wavelet packet decomposition could be applied in future research to obtain detail levels more fitting to the frequency features of the AF signal [57]. In addition, another future goal is to perform multiclass classification and/or AHI estimation through other advanced techniques of pattern recognition, such as deep-learning [58], and pediatric AF at-home recordings. Finally, the analysis proposed in this study could be used along with other PSG-derived signals, such as thoracic or abdominal effort, to distinguish among obstructive, central, and mixed apneic events in future research [15].

## 6. Conclusions

In this study, nocturnal AF of children has been characterized by means of features from wavelet analysis. We found that apneic events decrease the detail coefficients of the DWT associated to the normal breathing band (lower values of *M*_1*D*8_, *Max_D_*_8_, and *Min_D_*_8_), as well as the activity produced in the frequency range 0.1953–0.3906 Hz of AF (lower values of *E_D_*_8_). Wavelet analysis also revealed that OSA changes the frequency and energy distribution of AF signal, reducing its frequency components in the normal breathing band (higher values of *M*_3*D*8_ and *M*_4*D*8_) and generating more global irregularity in the signal (higher values of *WE*). Our study also found complementarity between DWT features from AF and *ODI*3. These findings allowed us to obtain a BY-MLP model with high diagnostic accuracy to estimate the AHI of mildly to moderately affected children, as well as an AdaBoost.M2 model particularly useful for classifying severe pediatric subjects. Therefore, we conclude that DWT can characterize the nocturnal AF, and that such approach could be jointly used with *ODI*3 to simplify the diagnosis of OSA in children.

## Figures and Tables

**Table 1:** Demographic and clinical characteristics of the different subject groups included during the training and testing phases of the study.

Characteristic	All	Training Group	Test Group
#Subjects	946	570 (60%)	376 (40%)
Age (years)	6 [6]	6 [5]	6 [6]
#Males	584 (61.7%)	339 (59.5%)	245 (65.2%)
BMI (kg/m^2^)	17.9 [6.2]	17.7 [6.7]	18.1 [6.0]
AHI (e/h)	3.8 [7.8]	4.2 [8.3]	3.3 [6.4]
#No-OSA	163 (17.2%)	91 (16.0%)	72 (19.1%)
#Mild OSA	386 (40.8%)	223 (39.1%)	163 (43.4%)
#Moderate OSA	172 (18.2%)	111 (19.5%)	61 (16.2%)
#Severe OSA	225 (23.8%)	145 (25.4%)	80 (21.3%)

The characteristics are presented as median [interquartile range] or number (%). BMI = body mass index, AHI = apnea-hypopnea index, OSA = obstructive sleep apnea.

**Table 2:** Statistical analysis of the extracted features in the training group.

Feature	No-OSA		Mild OSA		Moderate OSA		Severe OSA		RHO	*p*-Value
	Median	IQR	Median	IQR	Median	IQR	Median	IQR		
*M*_1*D*8_	2.62	0.97	2.46	0.86	2.29	1.03	1.67	1.08	−0.4024	<<0.01
*M*_2*D*8_	2.64	1.27	2.34	1.20	2.27	1.34	1.61	1.28	−0.3058	<0.01
*M*_3*D*8_	0.25	0.69	0.29	0.54	0.50	0.79	1.05	1.06	0.4413	<<0.01
*M*_4*D*8_	2.87	2.76	2.98	1.96	3.58	2.92	5.32	4.34	0.3666	<0.01
*Max_D_*_8_	6.67	1.09	6.61	0.99	6.59	1.36	6.21	1.44	−0.1662	<0.01
*Min_D_*_8_ (10^−3^)	2.87	0.84	2.60	0.93	2.52	1.14	1.86	1.06	−0.4154	<<0.01
*E_D_*_8_ (10^3^)	2.68	1.56	2.36	1.41	2.19	1.67	1.33	1.44	−0.3809	<0.01
*WE*	0.26	0.04	0.25	0.04	0.26	0.05	0.28	0.05	0.2793	<0.01
*ODI*3	1.16	2.02	2.21	3.24	4.36	5.98	14.28	18.68	0.6979	<<0.01

IQR = Interquartile range; RHO = Spearman’s correlation between the feature and the AHI; *p*-value = result of Kruskal-Wallis test after Bonferroni correction; *p*-value < 10^−19^ is denoted as <<0.01.

**Table 3:** Diagnostic evaluation of the proposed models and *ODI*3.

**AHI cut-off = 1 e/h**								
**Model**	**Se (%)** **[95%CI]**	**Sp (%)** **[95%CI]**	**Acc (%)** **[95%CI]**	**PPV (%)** **[95%CI]**	**NPV (%)** **[95%CI]**	**LR+** **[95%CI]**	**LR-** **[95%CI]**	**kappa_2_**
AB^AF^	79.89 ^a,b,c,d^ [77.10,82.46]	47.24 ^a,b,c,d^ [39.35,54.83]	73.61 ^a,b,c,d^ [70.86,76.27]	86.43 ^a,b,c,d^ [83.88,88.85]	35.62 ^a,b,c,d^ [29.29,41.47]	1.52 ^a,b,c,d^ [1.36,1.88]	0.43 ^a,b,c,d^ [0.36,0.57]	0.2395 ^a,b,c,d^ [0.1692,0.3059]
AB^AF,*ODI*3^	80.26 ^a,e,f,g^ [77.63,83.05]	68.07 ^a,e,f,g^ [61.85,74.49]	77.97 ^a,e,f,g^ [75.54,80.51]	91.45 ^a,e,f,g^ [89.31,93.30]	44.94 ^a,e,f,g^ [38.97,51.32]	2.56 ^a,e,f,g^ [2.20,3.47]	0.29 ^a,e,f,g^ [0.25,0.35]	0.4040 ^a,e,f,g^ [0.3406,0.4677]
BY-MLP^AF^	100.00 ^b,e,h,i^ [100.00, 100.00]	0.00 ^b,e,h,i^ [0.00, 0.00]	80.85 ^b,e,h,i^ [78.47,83.18]	80.85 ^b,e,h,i^ [78.47,83.18]	ND ^b,e,h,i^	1.00 ^b,e,h,i^ [1.00, 1.00]	ND ^b,e,h,i^	0.00 ^b,e,h,i^ [0.00, 0,00]
BY-MLP^AF,*ODI*3^	91.16 ^c,f,h,j^ [89.14,93.02]	43.28 ^c,f,h,j^ [36.45,50.59]	81.96 ^c,f,h,j^ [79.46,84.25]	87.18 ^c,f,h,j^ [84.93,89.18]	53.55 ^c,f,h,j^ [45.68,61.85]	1.62 ^c,f,h,j^ [1.46,1.92]	0.21 ^c,f,h,j^ [0.16,0.29]	0.3696 ^c,f,h,j^ [0.2944,0.4413]
*ODI*3	59.78 ^d,g,i,j^ [56.66,63.32]	86.06 ^d,g,i,j^ [80.83,90.79]	64.81 ^d,g,i,j^ [61.89,67.97]	94.79 ^d,g,i,j^ [92.83,96.64]	33.68 ^d,g,i,j^ [29.46,38.12]	4.59 ^d,g,i,j^ [3.52,10.83]	0.47 ^d,g,i,j^ [0.42,0.52]	0.2875 ^d,g,i,j^ [0.2424,0.3422]
**AHI cut-off = 5 e/h**								
**Model**	**Se (%)** **[95%CI]**	**Sp (%)** **[95%CI]**	**Acc (%)** **[95%CI]**	**PPV (%)** **[95%CI]**	**NPV (%)** **[95%CI]**	**LR+** **[95%CI]**	**LR-** **[95%CI]**	**kappa_2_**
AB^AF^	74.43 ^a,b,c,d^ [70.10,79.12]	47.18 ^a,b,c,d^ [43.31,51.21]	57.46 ^a,c,d^ [54.40,60.51]	45.81 ^a,c,d^ [42.12,49.93]	75.57 ^a,b,c,d^ [71.57,79.90]	1.42 ^a,c,d^ [1.30,1.58]	0.54 ^a,b,c,d^ [0.44,0.65]	0.1928 ^a,c,d^ [0.1408,0.2467]
AB^AF,*ODI*3^	68.03 ^a,e,f,g^ [63.10,72.79]	90.28 ^a,e,f,g^ [87.94,92.45]	81.91 ^a,e,f^ [79.50,84.36]	80.78 ^a,e,f,g^ [76.22,85.18]	82.49 ^a,e,f,g^ [79.67,85.32]	7.18 ^a,e,f,g^ [5.91,11.14]	0.35 ^a,e,f,g^ [0.30,0.41]	0.6009 ^a,e,f^ [0.5497,0.6540]
BY-MLP^AF^	77.25 ^b,e,h,i^ [73.13,81.50]	45.05 ^b,e,h,i^ [41.40,49,13]	57.14 ^e,h,i^ [54.20,60.27]	45.82 ^e,h,i^ [42.03,49.81]	76.89 ^b,e,h,i^ [72.83,81.24]	1.41 ^e,h,i^ [1.30,1.57]	0.50 ^b,e,h,i^ [0.41,0.62]	0.1967 ^e,h,i^ [0.1475,0.2481]
BY-MLP^AF,*ODI*3^	79.32 ^c,f,h,j^ [74.90,83.50]	83.83 ^c,f,h,j^ [80.92,86.61]	82.14 ^c,f,h,j^ [79.84,84.40]	74.57 ^c,f,h,j^ [70.37,79.04]	87.17 ^c,f,h,j^ [84.52,89.91]	4.97 ^c,f,h,j^ [4.28,6.52]	0.25 ^c,f,h,j^ [0.20,0.30]	0.6221 ^c,f,h,j^ [0.5754,0.6696]
*ODI*3	69.45 ^d,g,i,j^ [64.63,74.16]	89.38 ^d,g,i,j^ [86.91,91.68]	81.88 ^d,i,j^ [79.54,84.25]	79.79 ^d,g,i,j^ [75.04,83.97]	83.01 ^d,g,i,j^ [80.30,85.84]	6.68 ^d,g,i,j^ [5.60,10.17]	0.34 ^d,g,i,j^ [0.29,0.40]	0.6024 ^d,i,j^ [0.5509,0.6553]
**AHI cut-off = 10 e/h**								
**Model**	**Se (%)** **[95%CI]**	**Sp (%)** **[95%CI]**	**Acc (%)** **[95%CI]**	**PPV (%)** **[95%CI]**	**NPV (%)** **[95%CI]**	**LR+** **[95%CI]**	**LR-** **[95%CI]**	**kappa_2_**
AB^AF^	41.06 ^a,b,c,d^ [34.66,47.67]	85.52 ^a,b,c,d^ [83.13,87.83]	76.07 ^a,b,c,d^ [73.51,78.39]	43.30 ^a,b,c,d^ [36.89,50.53]	84.28 ^a,b,c,d^ [81.92,86.60]	2.86 ^a,b,c,d^ [2.36,3.80]	0.69 ^a,b,c,d^ [0.61,0.77]	0.2697 ^a,b,c,d^ [0.2040,0.3363]
AB^AF,*ODI*3^	72.37 ^a,e,f,g^ [66.59,77.90]	95.99 ^a,e,f,g^ [94.60,97.31]	90.99 ^a,e,f,g^ [89.29,92.61]	83.01 ^a,e,f,g^ [77.43,88.45]	92.76 ^a,e,f,g^ [91.03,94.44]	18.99 ^a,e,f,g^ [14.60,51.76]	0.29 ^a,e,f,g^ [0.23,0.35]	0.7159 ^a,e,g^ [0.6605,0.7677]
BY-MLP^AF^	50.00 ^b,e,h,i^ [42.96,56.68]	75.96 ^b,e,h,i^ [73.18,78.80]	70.47 ^b,e,h,i^ [67.77,73.07]	35.97 ^b,e,h,i^ [30.57,41.70]	84.86 ^b,e,h,i^ [82.28,87.30]	2.10 ^b,e,h,i^ [1.77,2.55]	0.66 ^b,e,h,i^ [0.57,0.76]	0.2271 ^b,e,h,i^ [0.1623,0.2886]
BY-MLP^AF,*ODI*3^	74.85 ^c,f,h,j^ [68.75,80.51]	95.00 ^c,f,h,j^ [93.42,96.46]	90.69 ^c,f,h,j^ [88.87,92.47]	80.04 ^c,f,h,j^ [74.53,85.78]	93.32 ^c,f,h,j^ [91.66,94.91]	15.60 ^c,f,h,j^ [12.23,30.31]	0.26 ^c,f,h,j^ [0.21,0.33]	0.7141 ^c,h,j^ [0.6570,0.7660]
*ODI*3	81.05 ^d,g,i,j^ [75.71,86.12]	88.58 ^d,g,i,j^ [86.34,90.76]	87.00 ^d,g,i,j^ [84.93,89.06]	65.84 ^d,g,i,j^ [60.31,71.74]	94.55 ^d,g,i,j^ [93.01,96.10]	7.23 ^d,g,i,j^ [6.10,9.98]	0.21 ^d,g,i,j^ [0.16,0.27]	0.6422 ^d,g,i,j^ [0.5894,0.6956]

Se = sensitivity; Sp = specificity; Acc = accuracy; PPV = positive predictive value; NPV = negative predictive value; LR+ = positive likelihood ratio; LR- = negative likelihood ratio; kappa_2_ = Cohen’s kappa of two-class; 95%CI = 95% confidence interval; ND = Non defined; *ODI*3 = 3% oxygen desaturation index; AB^AF^ = Adaboost.M2 fed with optimal wavelet features from AF; AB^AF,*ODI*3^ = Adaboost.M2 fed with optimal wavelet features from AF and *ODI*3; BY-MLP^AF^ = Bayesian multi-layer perceptron fed with optimal wavelet features from AF; BY-MLP^AF,*ODI*3^ = Bayesian multi-layer perceptron fed with optimal wavelet features from AF and *ODI*3, ^a^ Significant differences (*p*-value < 0.001) between AB^AF^ and AB^AF,*ODI*3^; ^b^ Significant differences (*p*-value < 0.001) between AB^AF^ and BY-MLP^AF^; ^c^ Significant differences (*p*-value < 0.001) between AB^AF^ and BY-MLP^AF,*ODI*3^; ^d^ Significant differences (*p*-value < 0.001) between AB^AF^ and *ODI*3; ^e^ Significant differences (*p*-value < 0.001) between AB^AF,*ODI*3^ and BY-MLP^AF^; ^f^ Significant differences (*p*-value < 0.001) between AB^AF,*ODI*3^ and BY-MLP^AF,*ODI*3^; ^g^ Significant differences (*p*-value < 0.001) between AB^AF,*ODI*3^ and *ODI*3; ^h^ Significant differences (*p*-value < 0.001) between BY-MLP^AF^ and BY-MLP^AF,*ODI*3^; ^i^ Significant differences (*p*-value < 0.001) between BY-MLP^AF^ and *ODI*3; ^j^ Significant differences (*p*-value < 0.001) between BY-MLP^AF,*ODI*3^ and *ODI*3.

**Table 4:** Global diagnostic metrics of the proposed models and *ODI*3.

	kappa_4_ [95%CI]	Acc_4_ (%) [95%CI]
AB^AF^	0.1126 [0.0796,0.1466] ^a,b,c,d^	30.52 [27.90,33.37] ^a,b,c,d^
AB^AF,*ODI*3^	0.4021 [0.3605,0.4463] ^a,e,f,g^	57.46 [54.47,60.60] ^a,e,f^
BY-MLP^AF^	0.0664 [0.0342,0.1004] ^b,e,h,i^	32.53 [29.87,35.20] ^b,e,h,i^
BY-MLP^AF,*ODI*3^	0.4088 [0.3637,0.4493] ^c,f,h,j^	58.57 [55.36,61.47] ^c,f,h,j^
*ODI*3	0.3826 [0.3362,0.4258] ^d,g,i,j^	57.23 [53.95,60.22] ^d,i,j^

kappa_4_ = Cohen’s kappa of four-class; Acc_4_ = four-class accuracy; 95%CI = 95% confidence interval; *ODI*3 = 3% oxygen desaturation index; AB^AF^ = Adaboost.M2 fed with optimal wavelet features from AF; AB^AF,*ODI*3^ = Adaboost.M2 fed with optimal wavelet features from AF and *ODI*3; BY-MLP^AF^ = Bayesian multi-layer perceptron fed with optimal wavelet features from AF; BY-MLP^AF,*ODI*3^ = Bayesian multi-layer perceptron fed with optimal wavelet features from AF and *ODI*3; ^a^ Significant differences (*p*-value < 0.001) between AB^AF^ and AB^AF,*ODI*3^; ^b^ Significant differences (*p*-value < 0.001) between AB^AF^ and BY-MLP^AF^; ^c^ Significant differences (*p*-value < 0.001) between AB^AF^ and BY-MLP^AF,*ODI*3^; ^d^ Significant differences (*p*-value < 0.001) between AB^AF^ and *ODI*3; ^e^ Significant differences (*p*-value < 0.001) between AB^AF,*ODI*3^ and BY-MLP^AF^; ^f^ Significant differences (*p*-value < 0.001) between AB^AF,*ODI*3^ and BY-MLP^AF,*ODI*3^; ^g^ Significant differences (*p*-value < 0.001) between AB^AF,*ODI*3^ and *ODI*3; ^h^ Significant differences (*p*-value < 0.001) between BY-MLP^AF^ and BY-MLP^AF,*ODI*3^; ^i^ Significant differences (*p*-value < 0.001) between BY-MLP^AF^ and *ODI*3; ^j^ Significant differences (*p*-value < 0.001) between BY-MLP^AF,*ODI*3^ and *ODI*3.

**Table 5:** Comparison with other studies of the literature.

Study	Nº Subjects (Total Dataset/Test Set)	Signal	Methods (Analysis/Selection/Classification)	AHI cut-off (e/h)	Se (%)	Sp (%)	PPV (%)	NPV (%)	LR+	LR-	Acc (%)
Shouldice et al. (2004) [10]	50/25	ECG	Temporal and spectral analysis/–/ QDA	1	85.70	81.80	85.70	81.80	4.71	0.18	84.00
Gil et al. (2010) [8]	21/21	PPG	Analysis of HRV, PTTV, and DAP events/Wrap method/LDA	5	75.00	85.70	-	-	5.24 *	0.29 *	80.00
Dehkordi et al. (2016) [9]	146/146	PPG	Temporal, spectral, and detrended fluctuation analysis/LASSO/LASSO	5	76.00	68.00	-	-	2.38 *	0.35 *	71.00
Hornero et al. (2017) [13]	4,191/3,602	SpO_2_	Statistical, spectral, non-linear analysis, and *ODI*3/FCBF/ MLP	1	84.02	53.19	81.64	57.34	1.79	0.30	75.15
				5	68.16	87.19	68.62	86.95	5.32	0.37	81.65
				10	68.66	94.07	67.68	94.31	11.58	0.33	90.17
Vaquerizo-Villar et al. (2018) [25]	981/392	SpO_2_	Statistical, spectral, wavelet analysis, and *ODI*3/FCBF/SVM	5	71.90	91.10	83.80	84.50	14.60	0.31	84.00
Xu et al. (2019) [47]	432/432	SpO_2_	*ODI*3 and 3rd statistical moment of the spectral band of interest/FCBF/MLP	1	95.34	19.10	81.96 *	51.52 *	1.18	0.25	79.63
				5	77.78	80.46	72.28 *	84.68 *	3.99	0.27	79.40
				10	73.53	92.73	75.76 *	91.89 *	10.07	0.29	88.19
Garde et al. (2019) [14]	207/207	SpO_2_ PRV	Temporal and spectral analysis/Stepwise-selection/LR	1	80.00	65.00	-	-	2.29 ^*^	0.31 ^*^	75.00
				5	85.00	79.00	-	-	4.05 ^*^	0.19 ^*^	82.00
				10	82.00	91.00	-	-	9.11 ^*^	0.20 ^*^	89.00
Barroso-García et al. (2020) [17]	946/376	AF *ODI*3	Recurrence quantification analysis and *ODI*3_/_FCBF/BY-MLP	1	97.70	22.22	84.14	69.57	1.26	0.10	83.24
				5	78.72	78.30	68.52	85.98	3.63	0.27	78.46
				10	78.75	94.26	78.75	94.26	13.71	0.23	90.96
Jiménez- García et al. (2020) [12]	974/390	AF SpO_2_	Statistical, non-linear, spectral analysis, and *ODI*3/FCBF / Multiclass AdaBoost.M2 with LDA	1	92.06	36.00	85.80	51.92	1.44	0.22	81.28
				5	76.03	85.66	76.03	85.66	5.30	0.28	82.05
				10	62.65	97.72	88.14	90.63	27.48	0.38	90.26
Barroso-García et al. (2021) [56]	946/376	AF *ODI*3	Bispectral analysis and *ODI*3_/_FCBF/MLP	1	98.03	15.27	83.01	65.01	1.16	0.14	82.16
				5	81.56	83.00	74.17	88.25	4.85	0.22	82.49
				10	72.29	94.98	79.58	92.69	15.01	0.29	90.15
This study	946/376	AF *ODI*3	Wavelet analysis and *ODI*3_/_FCBF/Multiclass AdaBoost.M2 with decision trees	1	80.26	68.07	91.45	44.94	2.56	0.29	77.97
				5	68.03	90.28	80.78	82.49	7.18	0.35	81.91
				10	72.37	95.99	83.01	92.76	18.99	0.29	90.99
			Wavelet analysis and *ODI*3_/_FCBF/BY-MLP	1	91.16	43.28	87.18	53.55	1.62	0.21	81.96
				5	79.32	83.83	74.57	87.17	4.97	0.25	82.14
				10	74.85	95.00	80.04	93.32	15.60	0.26	90.69

QDA = Quadratic discriminant analysis, HRV = Heart rate variability, PTTV = Pulse transit time variability, DAP = Decreases in amplitude fluctuations of the PPG signal, LDA = Linear discriminant analysis, LASSO = Least absolute shrinkage and selection operator, *ODI*3 = 3% oxygen desaturation index, FCBF = Fast correlation based filter, MLP = Multi-Layer perceptron neural network, FSLR = Forward stepwise logistic regression, LR = Logistic regression model, SVM = Support vector machine, PRV = Pulse rate variability, BY-MLP = Multi-Layer perceptron neural network with Bayesian approach. * Computed from reported data.
